# Isolation of endothelial progenitor cells from human adipose tissue

**DOI:** 10.1038/s41366-025-01884-5

**Published:** 2025-08-23

**Authors:** Cristina Caccioppoli, Rossella D’Oria, Valentina Annamaria Genchi, Giuseppe Palma, Valentina Andrulli Buccheri, Isabella Calderoni, Carmen Tedesco, Carmela Colabufo, Nicola Marrano, Giuseppina Biondi, Nada Chaoul, Antonio Braun, Angela Pezzolla, Angelo Cignarelli, Annalisa Natalicchio, Luigi Laviola, Francesco Giorgino, Sebastio Perrini

**Affiliations:** 1 Section of Internal Medicine, Endocrinology, Andrology and Metabolic Diseases, Department of Precision and Regenerative Medicine and Ionian Area, University of Bari Aldo Moro, Bari, Italy; 2 Section of Medical Oncology, Interdisciplinary Department of Medicine, University of Bari Aldo Moro, Bari, Italy; 3 GVM Care & Research, Bari, Italy; 4 Section of Video-Laparoscopic Surgery, Department of Precision and Regenerative Medicine and Ionian Area, University Aldo Moro of Bari, Bari, Italy; 5 Section of Endocrinology, Department of Medicine and Surgery, LUM University, Casamassima, BA Italy

**Keywords:** Biological techniques, Cell biology

## Abstract

**Background:**

Endothelial progenitor cells (EPCs) play an important role in angiogenic responses in multiple tissues and mediate a coordinate augmentation of the capillary network as adipose tissue (AT) expands in response to positive energy balance. However, the isolation and culture of EPCs from human AT has proven difficult so far. Here, we report the isolation and characterization of EPCs from human AT (AT-EPCs).

**Methods:**

Omental and subcutaneous AT specimens (approximately 1-2 g) were obtained during abdominal surgery. Following AT digestion with collagenase, both the filtered (SVF-I) and unfiltered (SVF-II) stromal vascular fractions (SVF) of AT were used. Expression of endothelial markers, such as CD31 and VE-Cadherin, was analyzed by using flow cytometry. Both SVF-I and SVF-II fractions were used for magnetic-based enrichment of endothelial cells using anti-human CD31 beads. Immunofluorescence staining, immunoblotting, and quantitative real-time PCR were performed to analyze expression of endothelial markers. Functional assays, including matrigel-based capillary-like tube formation assay and acetylated LDL uptake assays, were also performed.

**Results:**

CD31 and VE-Cadherin were more expressed in SVF-II than SVF-I. CD31+ cells from SVF-II exhibited an endothelial-like cobblestone morphology. The CD31+ fraction also expressed Von Willebrand Factor (vWF) and VE-Cadherin. High mRNA levels of E-selectin, e-NOS, VEGFR, and CD34 were found in CD31+ cells, and E-selectin and e-NOS proteins were readily detectable. In addition, CD31+ cells were able to form tubes and incorporate acetylated LDL in vitro.

**Conclusions:**

Large amounts of AT-EPCs with distinct functional properties can be isolated from omental and subcutaneous adipose tissue.

## Introduction

Endothelial progenitor cells (EPCs) are defined as a population of new cells released into the peripheral blood by the bone marrow, able to differentiate into mature endothelial cells (ECs) and involved in angiogenesis in the setting of hypoxia, ischemia, injury, or tumor formation [1–4]. Importantly, EPCs, with respect to ECs, are characterized by a higher proliferative potential and capacity to replace damaged endothelium [5]. For these reasons, EPCs are becoming important candidate cell sources for regenerative medicine [6], as demonstrated by numerous completed and ongoing trials, which investigate their potential use in multiple disease conditions, including cerebral, myocardial, and renal ischemic injury, peripheral vascular disease, and diabetic ulcers [7–12]. However, the main problem preventing clinical applications of such a cellular source is the lack of an efficient protocol able to isolate adequate amounts of EPCs required for their use in regenerative medicine.

EPCs were first identified and isolated from adult peripheral blood by Asahara and colleagues [13]. However, it was later demonstrated that peripheral blood did not represent an ideal source of EPCs, because the amount of EPCs in the circulation is relatively low [14], and these cells maintain typical monocytic functions with little proliferative capacity [15]. EPCs have also been isolated from adult bone marrow, even though it is difficult to obtain them from this compartment [5, 16]. More recently, investigators have also explored the possibility of isolating EPCs from umbilical cord blood; these EPCs are characterized by a higher proliferative potential and are less immunogenic compared to those from adult peripheral blood [17–19]. Nevertheless, the use of EPCs isolated from umbilical cord blood in regenerative medicine also has limitations, such as their tumorigenic potential.

Recent evidence has shown that EPCs are also present in adipose tissue (AT) [20], an endocrine organ largely composed of mature adipocytes and stromal cells, the latter defined as the stromal vascular fraction (SVF) [21]. Specifically, the SVF contains multipotent mesenchymal stem/progenitor cells, which can differentiate into several lineages [22], including the EPCs [14]. The AT can potentially provide large amounts of autologous EPCs, as it is abundant in the human body, can be withdrawn through a minimally invasive procedure, and is relatively enriched in EPCs [14, 21]. The multi-parameter analysis of single cells in flow cytometry, using multiple cell surface markers such as markers for hematopoietic cells (CD45), immaturity (CD133), stemness (CD34), and endothelial commitment (KDR), has been used to identify EPCs in the cell sources described above [23]. However, it is particularly difficult to accurately identify EPCs with this methodology, as some of these markers are expressed by both EPCs and hematopoietic cells. More recently, Ravishankar et al. have reported a new method for the isolation of EPCs from human umbilical cord blood, including the use of a specialized endothelial medium supplemented with growth factors and subsequent characterization of the isolated cells by using immunoblotting and immunostaining [24]. EPCs were also obtained from the SVF of AT (lipoaspirate) following enzymatic digestion with collagenase (AT-EPCs) and were then cultured in specific culture media in a 2-step procedure [25]. AT-EPCs were analyzed for specific surface marker expression, including CD31 and VEGFR2, as well as for angiogenesis capability in vitro [25]. However, this procedure required a large amount of AT, making it relatively difficult to be performed. In this study, we present the isolation and characterization of AT-EPCs through a novel method that makes use of small AT amounts with high yield, allowing for their rapid isolation and expansion.

## Materials and methods

### AT biopsies

Omental and subcutaneous AT biopsies were obtained from 20 subjects with obesity (Supplementary Table 1) undergoing elective open-abdominal surgery. None of the patients had diabetes or severe systemic illness, and none was taking medications known to affect AT mass or metabolism. However, as it was not possible to take subcutaneous AT from all enrolled patients, most of the endothelial cell isolation experiments were performed using omental adipose tissue. The protocol was approved (approval 152/2012) by the Independent Ethical Committee at the Azienda Ospedaliero-Universitaria Policlinico Consorziale, University of Bari Aldo Moro, and all patients gave their informed consent. The work described has been carried out in accordance with WHO Guiding Principles on Human Cell, Tissue, and Organ Transplantation.

### Generation and culture of SVF-I and SVF-II from human AT and HUVECs

**Fig. 1:**
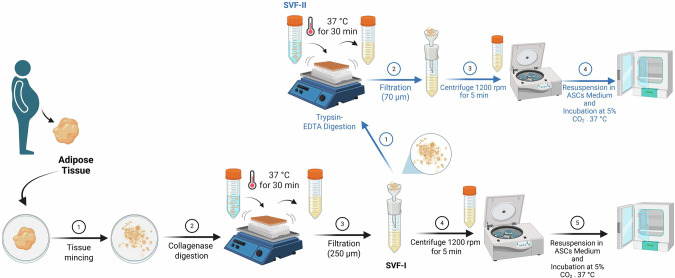
Protocol for isolation of endothelial progenitor cells from solid fractions of human adipose tissue. Fat tissue fragments (approximately 1–2 g) were minced and digested. The resulting material was filtered and the flow-through, termed SVF-I, was centrifuged. The obtained floating fraction was plated in the presence of culture medium specific for human ASC, as previously described [26]. The digestion product retained by cell strainer, termed SVF-II, was further digested, filtered, and the flow-through was centrifuged. The supernatant obtained was plated in the presence of culture medium specific for human ASC [26]. ASC adipose stem cells, SVF stromal vascular fraction. The figure was created using BioRender Software.

As shown in Fig. 1, AT fragments (approximately 1–2 g) were minced and digested using 1 mg/ml type I collagenase (Sigma-Aldrich, St. Louis, MO), with gentle shaking at 37 °C for 30 min. The resulting material was filtered through a 250-µm strainer, and the flow-through, termed SVF-I, was centrifuged at 1200 rpm for 5 min at room temperature. The obtained floating fraction was plated in culture medium for human adipose stem cells (ASCs), as described [26]. The digestion product retained by the cell strainer, referred to as SVF-II, was further digested using 0.25% trypsin/EDTA at 37 °C for 30 min. The resulting material was filtered through a 70-µm strainer, and the flow-through was centrifuged at 1200 rpm for 5 min. Similarly, the resulting supernatant was plated in culture medium for human ASCs [26]. We employed sequential filtration through 250 μm and 70 μm meshes to achieve a high-resolution, stepwise separation that minimizes contamination while maximizing the purity and homogeneity of the isolated cells. The 250 μm mesh—consistent with established protocols and our own work on human subcutaneous and visceral adipose-derived stem cells [26–28]—removes particles >250 μm, including cell aggregates, connective-tissue fragments, and debris. The novel 70 μm mesh then further refines the filtrate by retaining the smaller cells liberated from SVF-II by trypsin/EDTA digestion and by excluding any residual tissue fragments that passed through the first filter. [26–28]. All the reagents described were from Life Technologies (Inc., Invitrogen, Carlsbad, CA). HUVECs were obtained from ATCC and cultured as previously described [29].

### Flow cytometry

Cells were washed in PBS, fixed with 4% paraformaldehyde for 20 min, and incubated with the appropriate monoclonal antibodies [CD31- FITC (Catalog No: 560984, BD Biosciences, San Jose, CA); VE Cadherin-PE (Catalog No: 130-135-356, Miltenyi Biotec, Bergisch Gladbach, Germany)] for 1 h, and analyzed with a Navios Flow Cytometer (Beckman Coulter Inc., Brea, California, USA).

### Magnetic microbead-based enrichment of CD31+ cells from SVF-I and SVF-II

Cells obtained from SVF-I and SVF-II fractions were detached using Trypsin-EDTA, pelleted at 272 x g for 5 min at room temperature, resuspended in PBS, 0.5% BSA, 2 mM EDTA, filtered through a 30-µm cell strainer, and counted. Then, the cell suspensions were used for magnetic microbead-based enrichment of endothelial cells using a human CD31 MicroBead Kit (Miltenyi Biotec, Bergisch Gladbach, Germany) according to the manufacturer’s instructions. Briefly, cells were resuspended to a maximum concentration of 1 × 10^7^ cells per 60 µl of M131 medium (Gibco, ThermoFisher, Waltham, MO, USA). Then, 20 μL of FcR blocking reagent was added to the cells, which were then incubated with 20 μL of CD31 microbeads for 15 min at 4 °C. At the end of the incubation, 1 mL of M131 medium was added to the cell suspension, which was subsequently centrifuged at 300 × g for 3 min, resuspended in 1 ml of M131 medium, and subjected to separation in the magnetic field of a MACS separator prewashed with 3 mL of M131 medium. After the cell suspension was applied onto the column, the column was washed three times with 3 ml of M131 medium, and unlabeled cells (CD31− cells) passed through and were centrifugated at 1200 rpm for 5 min at room temperature and plated in the presence of culture medium for human ASCs [26]. Then, the column was removed from the magnetic separator and placed on a suitable collection tube, and magnetically labeled CD31+ cells were eluted from the column by pipetting 5 ml of M131 medium and firmly pushing a plunger into the column. CD31+ cells were plated in cell culture dishes pretreated with attachment factor (Inc. Invitrogen, Carlsbad, CA) in the presence of M131 medium supplemented with the microvascular growth factor (MVGF), 1%NEM/NEAA, 1% penicillin/streptomycin, and 5% FBS (Inc. Invitrogen, Carlsbad, CA).

### Detection of von Willebrand factor and VE-Cadherin by immunofluorescence

Cells grown to 80% confluence on coverslips were fixed in ice-cold 4% paraformaldehyde for 15 min and permeabilized in PBS/0.1% Triton X-100 for 15 min. Samples were incubated with a primary monoclonal antibody against von Willebrand Factor (Catalog No: F3520) or VE-Cadherin (Catalog No: V1514) (1:80; both from Sigma Aldrich, St. Louis, MO) in PBS/3% BSA overnight at 4 °C, and then with a secondary Alexa (488) Fluor anti-rabbit goat antibody (Catalog No: Cat # A-11008) or a secondary Alexa (488) Fluor anti-mouse goat antibody (Catalog No: Cat # A-11001) (1:2000; Molecular Probes, Eugene, OR), respectively, for 1 h at 25 °C. Nuclei were stained with TO-PRO-3 (1:3000; Molecular Probes). Micrographs were acquired via a Leica TCS SP2 laser scanning spectral confocal microscope (Leica Microsystems) at the same magnification.

### Immunoblotting

Experimental cells were rapidly washed with Ca^2+^/Mg^2+^-free phosphate-buffered saline (PBS) and then mechanically detached in ice-cold lysis buffer containing 50 mmol/L HEPES pH 7.5, 150 mmol/L NaCl, 1 mmol/L MgCl_2_, 1 mmol/L CaCl_2_, 4 mmol/L EDTA, 1% Triton X-100, 10% glycerol, 50 mmol/L NaF, and 10 mmol/L NaPP, supplemented with phosphatase and protease inhibitors (Roche, Mannheim, Germany). Immunoblotting was carried out as previously described [27, 30]. Polyclonal antibody against E-Selectin (Catalog No: sc-137054) was purchased from Santa Cruz Biotechnology, Inc. (TX, USA); monoclonal antibody against e-NOS (Catalog No: 610297) was from BD Bioscience (CA, USA).

### Gene expression analysis by quantitative real-time PCR

Total RNA was purified using the RNeasy Mini Kit (Qiagen, Hilden, Germany), as previously described [26, 27, 31]. Total RNA (500 ng) was used for cDNA synthesis using the High-Capacity cDNA Reverse Transcription Kit (Applied Biosystems, Weiterstadt, Germany). PCRs were carried out in CFX ConnectTM (Bio-Rad) under the following conditions: polymerase activation and DNA denaturation at 95 °C for 30 sec, denaturation at 95 °C for 2 sec, and annealing/extension at 60 °C for 30 sec (40 cycles). Relative gene expression levels were determined by analyzing the changes in SYBR green fluorescence during quantitative PCR using the ΔCt method. The mRNA level of each gene was normalized using 18S as the internal control. All primers were designed using Primer Express 3.0 (Applied Biosystems; Supplementary Table 2).

### Geltrex-based capillary-like tube formation assay

HUVECs, CD31+ cells, and CD31- cells from SVF-II were cultured in Medium 200 supplemented with 1% antibiotics, 1% NEM/NEAA, and large vessel endothelial supplement (LVES) (Gibco, ThermoFisher, Waltham, MO, USA) until confluence. 200 μL of Geltrex™ LDEV-Free Reduced Growth Factor Basement Membrane Matrix (ThermoFisher, Waltham, MO, USA) was added to a 24-well plate and allowed to solidify at 37 °C for 30 min. 6 × 10^4^ cells were seeded onto the coated plates and incubated for 16 h to allow the formation of tubes [14]. Three representative images were recorded from each well using an optical microscope. Quantification of the tube number was performed using CellProfiler (v.4.2.8).

### Assessment of acetylated LDL uptake

HUVECs, CD31+ cells, and CD31- cells from SVF-II were plated onto glass slides in a 6-well plate and cultured until 70–80% confluence. The cells were incubated with 15 µg/ml Dil-acetylated-low-density lipoprotein (Dil AcLDL, Life Technologies, Inc., Invitrogen, Carlsbad, CA) in culture medium at 37 °C for 4 h [14]. Then, cells were washed twice with PBS 1X and fixed with 4% paraformaldehyde for 15 min. Nuclei were stained with TO-PRO-3 (1:3000; Molecular Probes). Micrographs were acquired via a Leica TCS SP2 laser scanning spectral confocal microscope (Leica Microsystems) at the same magnification. Quantification of the red perinuclear signal was performed using CellProfiler (v.4.2.8).

### Statistical analyses

All data are presented as mean ± standard error of the mean of at least three independent experiments. Statistical analysis was performed by a Student t test. Significance was assumed at *p* value < 0.05.

## Results

### Characterization of SVF-I and SVF-II derived cells

**Fig. 2:**
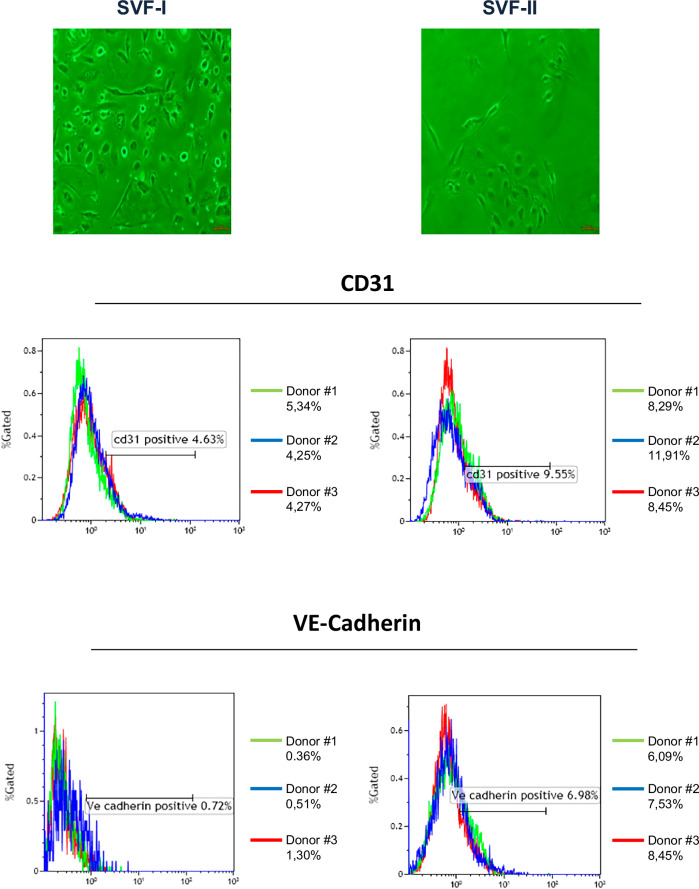
Flow cytometry characterization of SVF-I and SVF-II fractions from human adipose tissue. SVF-I (filtered) and SVF-II (unfiltered) fractions were studied by flow cytometry to analyze CD31 and VE-Cadherin expression. The results obtained from three different donors, as well as the average value, are shown. SVF stromal vascular fraction.

SVF-I (filtered) and SVF-II (unfiltered) cell fractions, obtained from omental AT specimens (Fig. 1), were studied by flow cytometry to analyze CD31 and VE-cadherin expression. As shown in Fig. 2, the proportion of CD31+ and VE-Cadherin+ cells was higher in SVF-II as compared to SVF-I (SVF-I mean values: CD31 4.63%, VE-Cadherin 0.72%; SVF-II mean values: CD31 9.55%, VE-Cadherin 6.98%).

### Magnetic microbead-based enrichment based on CD31 of SVF-I and SVF-II

**Fig. 3:**
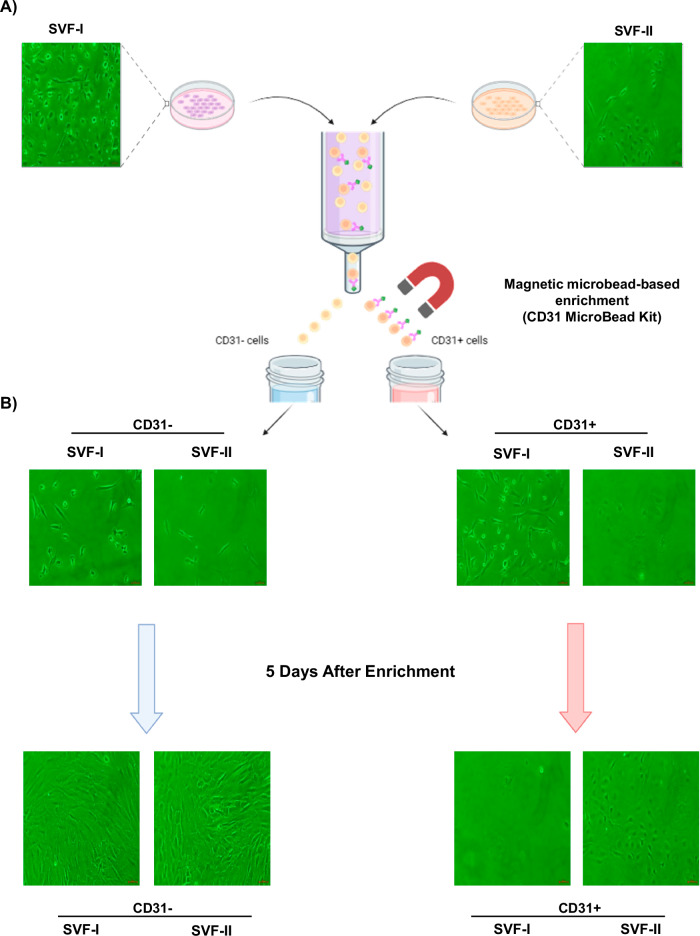
Magnetic microbead-based enrichment of CD31+ cells from SVF-I and SVF-II. **A** SVF-I and SVF-II underwent magnetic microbead-based enrichment based on CD31, and both fractions were observed by using light microscopy. **B** CD31+ and CD31- cells from enrichment were cultured, and after 5 days endothelial-like colonies with typical cobblestone morphologies were present only in the CD31+ cell cultures obtained from SVF-II. Scale bar, 10 µm. SVF stromal vascular fraction.

SVF-I and SVF-II, obtained from omental AT, underwent magnetic microbead-based enrichment based on CD31 expression, and both CD31+ cells and CD31- cell fractions were observed under light microscopy. CD31+ cells were identified both in SVF-I and SVF-II, although cells characterized by an endothelial-like cobblestone cell morphology were more abundant in SVF-II (Fig. 3A).

Both CD31+ and CD31- cells from SVF-I and SVF-II fractions were cultured. After 5 days of in vitro culture, endothelial-like colonies with typical cobblestone morphologies were present only in the CD31+ cell culture obtained from SVF-II (Fig. 3B). Conversely, CD31+ cells from SVF-I were almost absent (Fig. 3B). In contrast, CD31- cells from SVF-I and SVF-II exhibited a fibroblast-like morphology (Fig. 3B).

Magnetic microbead–based enrichment of CD31+ cells was carried out on both SVF-I and SVF-II fractions isolated also from subcutaneous adipose tissue (Supplementary Fig. 1). Endothelial-like colonies with characteristic cobblestone morphology appeared only in the CD31+ fraction from SVF-II (Supplementary Fig. 1B), whereas CD31- cells from both SVF-I and SVF-II exhibited a fibroblast-like morphology (Supplementary Fig. 1B).

### Analysis of endothelial cell markers in CD31+ and CD31- cells from SVF-II

**Fig. 4:**
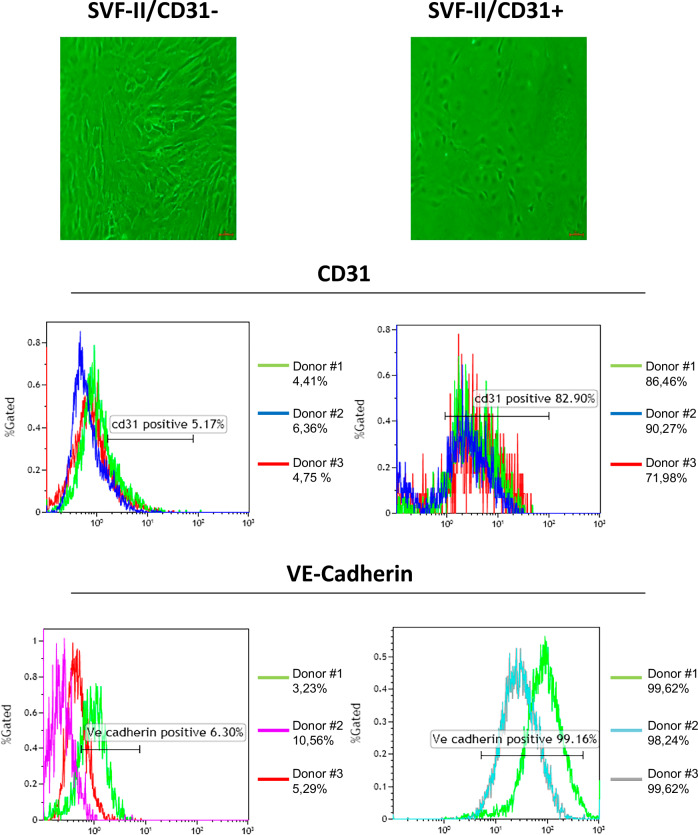
Flow cytometry characterization of CD31+ and CD31- cells from SVF-II. Both CD31+ and CD31- cells from microbead-based enrichment of SVF-II were cultured for 5 days and then underwent flow cytometry to analyze CD31 and VE-Cadherin expression. The results obtained from three different donors, as well as the average value, are shown. SVF stromal vascular fraction.

CD31+ and CD31- cells, obtained from the magnetic microbead-based enrichment based on CD31 of SVF-II, were cultured for 5 days and underwent flow cytometry analysis. This analysis showed that the proportion of CD31+ and VE-Cadherin+ cells was much higher, as expected, in the CD31+ compared to the CD31- cell population (mean values: CD31 82,90%, VE-Cadherin 99.16% vs. CD31 5.17%, VE-Cadherin 6.30%, respectively) (Fig. 4).

**Fig. 5:**
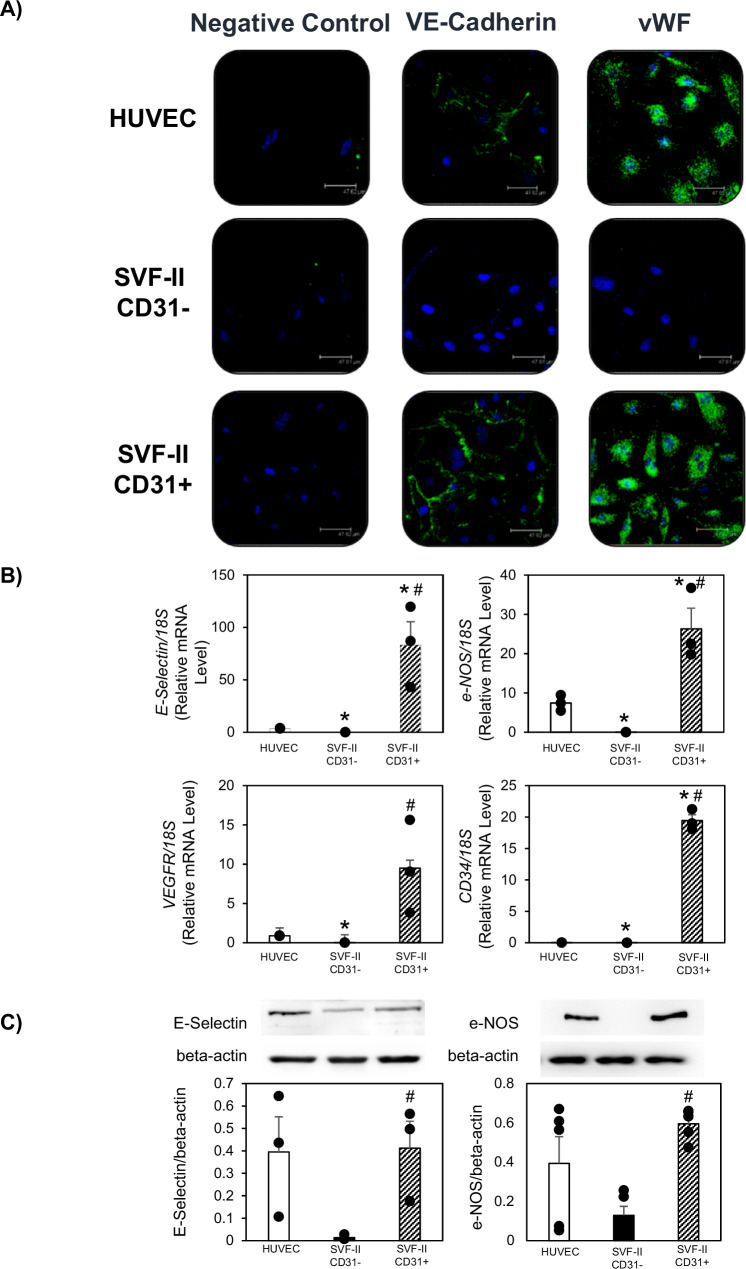
Expression of specific endothelial cell markers in CD31+ and CD31- cells from SVF-II. **A** Cells were fixed and incubated with Von Willebrand Factor (vWF) or VE-Cadherin antibodies, followed by Alexa Fluor (488) anti-rabbit antibody (green) to stain vWF or Alexa Fluor (488) anti-mouse antibody (green) to stain VE Cadherin, respectively. TO-PRO-3 was used to stain nuclei (blue). Scale bar: 47.62 mm. HUVECs were used as positive control. Negative control was represented by cells treated only with secondary antibody. **B** E-selectin, e-NOS, VEGFR, and CD34 mRNAs were analyzed in CD31+ and CD31- cells from SVF-II by quantitative reverse transcription PCR. All data are presented as mean ± standard error of the mean of three experiments, which were carried out using cells from different human donors. *, *p* < 0.05 vs HUVECs; #, *p* < 0.05 vs CD31- cells. HUVECs were used as positive control. 18S was used as loading control. **C** E-selectin and e-NOS proteins were analyzed in CD31+ and CD31- cells from SVF-II by immunoblotting. All data are presented as mean ± standard error of the mean of at three experiments for E-selectin, and five experiments for e-NOS, which were carried out using cells from different human donors. #, *p* < 0.05 vs CD31- cells. HUVECs were used as positive control. Beta-actin was used as loading control. HUVECs human umbilical vein endothelial cells, SVF stromal vascular fraction.

Antigenic characterization of CD31+ and CD31- cells from SVF-II was also performed by immunofluorescence staining, which confirmed that CD31+ cells expressed VE Cadherin, in contrast to the CD31- cell counterpart, and demonstrated also the presence of Von Willebrand Factor (vWF) in the CD31+ and not in the CD31- cell population (Fig. 5A).

CD31+ and CD31- cell populations from SVF-II were also studied for mRNA and protein expression of specific endothelial cell markers, such as E-selectin, e-NOS, VEGFR, and CD34, by both quantitative real-time PCR and immunoblotting. As shown in Fig. 5B, E-selectin, e-NOS, VEGFR, and CD34 mRNAs were expressed in CD31+ cells (*p* < 0.05 vs CD31- cells) and not in CD31- cells (*p* < 0.05 vs HUVECs) from SVF-II; interestingly, the mRNA levels of these endothelial cell markers were higher than in HUVECs (*p* < 0.05 vs HUVECs), which were used as a positive control (Fig. 5B). Moreover, E-selectin and e-NOS proteins were detectable at relatively high levels in CD31+ cells (*p* < 0.05 vs CD31- cells) and not in CD31- cells from SVF-II, and levels were comparable to those found in HUVECs (Fig. 5C).

mRNA and protein expression of the above specific endothelial cell markers were also identified in CD31+ cells isolated from SVF-II using subcutaneous AT obtained from different donors, thus demonstrating the feasibility of the protocol also using biopsies from the latter depot (Supplementary Fig. 2A, B).

### Effect of cell passage number on expression of VE-Cadherin in CD31+ cells from SVF-II

VE-Cadherin mRNA levels were evaluated in CD31+ cells from SVF-II cultured up to the fourth cell passage in the presence of medium specific for human EPCs [32]. As shown in Supplementary Fig. 3, CD31+ cells from SVF-II maintained VE-Cadherin mRNA expression (*p* < 0.05 vs CD31- cells), evaluated by quantitative real-time PCR Moreover, VE-Cadherin protein expression was also evaluated by immunoblotting and found to be stable up to the fourth cell passage (*p* < 0.05 vs CD31- cells) (Supplementary Fig. 3). HUVECs were used as positive control, CD31- cells as negative control.

### Assessment of tube formation in 3D scaffolds and acetylated LDL uptake

**Fig. 6:**
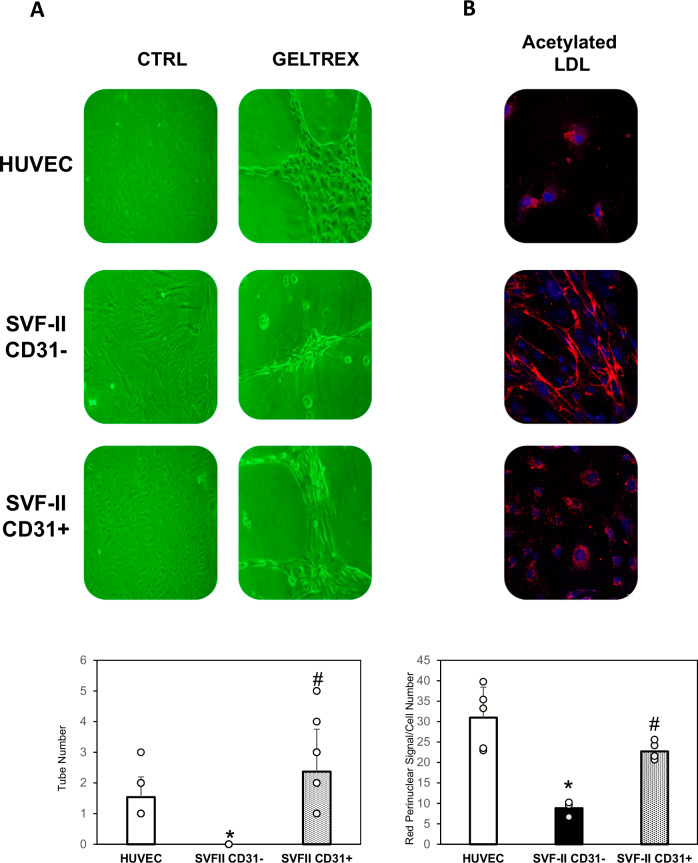
Functional characterization of CD31+ and CD31- cells from SVF-II. **A** Representative images obtained in light microscopy of tube formation of HUVECs, and SVF-II-derived CD31+ and CD31- cells grown on 3D scaffolds (Geltrex). The quantification of the tube number was performed using CellProfiler (v.4.2.8). *, *p* < 0.05 vs HUVECs; #, *p* < 0.05 vs CD31- cells. HUVECs were used as positive control. **B** Representative images obtained in fluorescence microscopy of acetylated LDL uptake (red) evaluated in HUVECs, and SVF-II-derived CD31+ and CD31- cells. TO-PRO-3 was used to stain nuclei (blue). The quantification of the red perinuclear signal was performed using CellProfiler (v.4.2.8). *, *p* < 0.05 vs HUVECs; #, *p* < 0.05 vs CD31- cells. HUVECs were used as positive control. HUVECs human umbilical vein endothelial cells, SVFs stromal vascular fraction.

Finally, we evaluated the capacity of tube formation in 3D scaffolds (Geltrex) and incorporate acetylated LDL, which represent indexes of functional endothelial cells. CD31+ cells from SVF-II were found to form capillary-like structures in Geltrex, comparably to HUVECs, which were used as a positive control, and differently from CD31- cells from SVF-II, as shown in Fig. 6A. As shown in Fig. 6B, LDL uptake was observed in CD31+ cells from SVF-II, as well as in HUVEC. Confocal microscopy studies show that CD31- cells from SVF-II also internalize LDL to a low extent. This finding likely reflects residual contamination by CD31+ cells, in agreement with the results presented in Figs. 2 and 4.

## Discussion

AT has long been considered only a metabolic reservoir of high-energy substrates, such as triglycerides, cholesterol, and lipid-soluble vitamins accumulating in adipocytes [25]. However, more recent results have clarified that it consists of multiple other cell types in addition to adipocytes, such as cell populations supporting tissue renewal [21, 33]. The SVF includes stromal cells, ASCs, which can differentiate into cells of several mesenchymal lineages, as well as cells from the microvasculature, including vascular endothelial cells and their progenitors, and vascular smooth muscle cells [22, 27, 34]. Although human EPCs have been generally isolated from adult peripheral blood, adult bone marrow, and umbilical cord blood [13, 16–18], these cells, characterized by an increased proliferative potential compared to mature cells, are also resident in adipose tissue.

In this study, we describe a new method for the isolation of AT-EPCs starting from small amounts of human abdominal AT. As detailed in Fig. 1, the first novel aspect of this protocol is the use of the unfiltered product obtained after digestion of solid AT with type I collagenase retained by a 250-µm cell strainer, which is discarded following standard protocols. Instead, this fraction, referred to as SVF-II, was further digested with trypsin-EDTA, filtered, centrifuged, and plated. It is worth noting that trypsin-EDTA and type I collagenase exert different effects on extracellular-matrix digestion, respectively. Collagenase can digest matrix fibers, acting by unwinding triple-helical collagen [35]. On the other hand, trypsin-EDTA can detach endothelial cells from vessels and extracellular matrix by disrupting focal adhesion joints mediated by integrin and cadherin adhesion molecules [36, 37]. Noteworthy, focal adhesion joints could be masked by matrix collagen fibers, whose triple helix structure is resistant to trypsin but not to collagenase. Therefore, the use of collagenase prior to trypsin digestion may improve isolation effectiveness by uncovering focal adhesion sites from collagen fibers and predisposing them to trypsinization, thus maximizing cell yield [38].

The second novel aspect is the use of smaller amounts of AT than those used in other protocols, i.e. 1–2 g compared to more than 5 g of human omental or subcutaneous tissue [39]. SVF-I and SVF-II were studied by flow cytometry to analyze CD31 and VE-Cadherin expression, two relevant markers used to identify EPCs [40]. As shown in Fig. 2, both SVF-I and SVF-II contained CD31+ and VE cadherin+ cells, although the proportion of these cells was higher in SVF-II compared to SVF-I. These data demonstrated that the AT- EPCs isolated with this new protocol have a similar phenotype to circulating EPCs. Van Pham et al. first demonstrated that human EPCs could also be obtained from AT. However, our protocol differs from that one as it does not rely on the different adhesion capabilities of mesenchymal stem cells (rapidly plastic adherent cells) versus AT-EPCs (slowly plastic adherent cells) [25].

Magnetic microbead-based enrichment of CD31+ cells from both SVF-I and SVF-II showed that CD31+ cells are present in both fractions, although SVF-II was enriched in cells with endothelial-like cell morphology (Fig. 3A). Additionally, after 5 days of in vitro culture, endothelial-like colonies with typical cobblestone morphologies were present only in the CD31+ cell culture obtained from SVF-II (Fig. 3B). Flow cytometry analysis, immunofluorescence staining, and analysis of mRNA and protein expression of specific endothelial cell markers confirmed the endothelial phenotype of CD31+ cells from the SVF-II fraction (Figs. 4 and 5). Specifically, similarly to circulating EPCs, CD31+ cells from SVF-II express hematopoietic and endothelial markers, including e-selectin, eNOS, VEGFR, and CD34 at levels like those found in HUVECs (Fig. 5B, C). Of note, EPCs do not express CD45, so assessing its presence during the isolation of EPCs allows contaminating cells from the blood or immune system to be excluded. In other words, the protocols published so far involve indirect EPC enrichment [41–43], unlike ours, which is based on direct EPC selection through magnetic microbead-based enrichment of CD31+ cells. This aspect represents a further novelty of our study.

Remarkably, with this new protocol, the expression of endothelial markers and the endothelial phenotype was maintained throughout the cell expansion process. Indeed, CD31+ cells from SVF-II cultured in the presence of medium specific for human EPCs were able to stably express VE-Cadherin, both mRNA and protein, up to the fourth cell passage (Supplementary Fig. 3), and this is in line with previous studies confirming that these cells maintain the endothelial phenotype in culture [24]. Moreover, assessment of the phenotypic stability of SVF-II–derived CD31+ cells was confined to passage 4, in keeping with their intended use in regenerative medicine. Notably, extending culture to four passages ( ≈ 4 weeks) enables robust expansion to yield a high total cell number suitable for therapeutic protocols. From passage 5 onward, these CD31+ cells exhibit the characteristic morphological hallmarks of cellular senescence. VE-Cadherin protein levels were even higher in CD31+ cells from SVF-II than in HUVECs, used as positive control, and almost absent in the counterpart CD31- (Supplementary Fig. 3).

CD31+ cells from SVF-II showed not only a similar phenotype to circulating human EPCs, but also significant functional properties, as they displayed potential to form new vessels shown by a capillary-like network formation in the Geltrex scaffold (Fig. 6A), and showed acetylated-LDL uptake in vitro (Fig. 6B), another well-known property of EPCs and endothelial cells [14, 25, 44–46]. The angiogenic properties shown by CD31+ cells from SVF-II suggest that AT-EPCs isolated with this protocol might be used as a promising cell source for pre-vascularization in tissue engineering. However, further studies are needed to investigate whether the new vascular network obtained from these cells can integrate with the host circulation following implantation [47].

The new protocol described here presents some advantages. First, human AT, being a widespread tissue, is easily available, and very small amounts of tissue are needed to isolate EPCs. Second, AT-EPCs can be isolated without sophisticated and expensive equipment and procedures, in contrast to other protocols largely based on the use of immunosorting [48]. In addition, in contrast with the isolation of EPCs from umbilical cord blood, a method also not based on immunosorting, no specific precautions are necessary after the centrifugation steps, including the lysis of erythrocytes, which could damage cells if longer incubation times are used. Third, autologous AT-EPCs might be potentially used for regenerative medicine and tissue engineering procedures without immune reactions [7]. Indeed, several preclinical and clinical studies support the therapeutic use of autologous EPCs mostly for cardiovascular repair, with potentially interesting results [23, 49]. When feasible, subcutaneous AT biopsies were also processed. Supplementary Figs. 1 and 2 demonstrate that the characterization of CD31+ cells isolated from subcutaneous SVF-II parallels that of CD31+ cells from omental SVF-II (Figs. 3 and 5B–C), underscoring the versatility of our isolation protocol. However, since subcutaneous AT biopsies could not be collected from all enrolled patients, the majority of experiments were conducted using omental AT biopsies alone.

Future studies will be necessary to further establish the pro-angiogenic potential of AT-EPCs isolated by this new protocol, involving for example investigation of VEGF release, one of the most important angiogenic factors [50], and extracellular vesicles for transfer of genetic information in injured tissues [51]. In addition, the role of AT-EPCs in neovascularization should be also assessed through *vivo* studies. Finally, the effects of co-culture of AT-EPCs and parenchymal cells could be investigated, as co-culture of endothelial cells with parenchymal cells might improve anastomosis between pre-vascularized networks and the host vasculature, as shown in several disease models [52].

This study presents some limitations. First, although both omental and subcutaneous adipose tissue (AT) were used for endothelial progenitor cell (EPC) isolation, the majority of experiments were conducted using omental AT. Consequently, further validation using a larger number of subcutaneous biopsies would be valuable to confirm the generalizability of the protocol across different fat depots. Second, the functional assays were restricted to in vitro models (tube formation and LDL uptake), with no in vivo validation to determine whether AT-derived EPCs (AT-EPCs) can effectively integrate into host vasculature or contribute to tissue neovascularization in regenerative settings. Future in vivo studies and clinical trials are warranted to better assess the therapeutic potential and translational relevance of AT-EPCs isolated using this method. Third, the study population was limited to individuals with obesity undergoing elective surgery; whether EPC yield or characteristics differ in individuals without obesity, or in the context of metabolic or inflammatory diseases, remains to be explored.

In conclusion, we have developed a new alternative and efficient protocol allowing for the isolation of large quantities of AT-EPCs from an easily accessible cell source, exhibiting specific functional properties, which could be investigated in disease states and used as autologous mesenchymal cells in regenerative medicine.

## Supplementary information

Supplementary Materials

## Data Availability

The datasets generated and/or analyzed during the current study are available from the corresponding author upon reasonable request. All data supporting the findings of this study are included in the article and its Supplementary Information files.
