# Supplementary material for: Isolation of endothelial progenitor cells from human adipose tissue
Source: Int J Obes (Lond). 2025 Aug 23;49(12):2462–72. doi: 10.1038/s41366-025-01884-5 (PMC12634424; doi:10.1038/s41366-025-01884-5)
Supplement: Supplementary file 1 — Supplementary Materials [file 41366_2025_1884_MOESM1_ESM.pdf]

Supplementary Figure 1

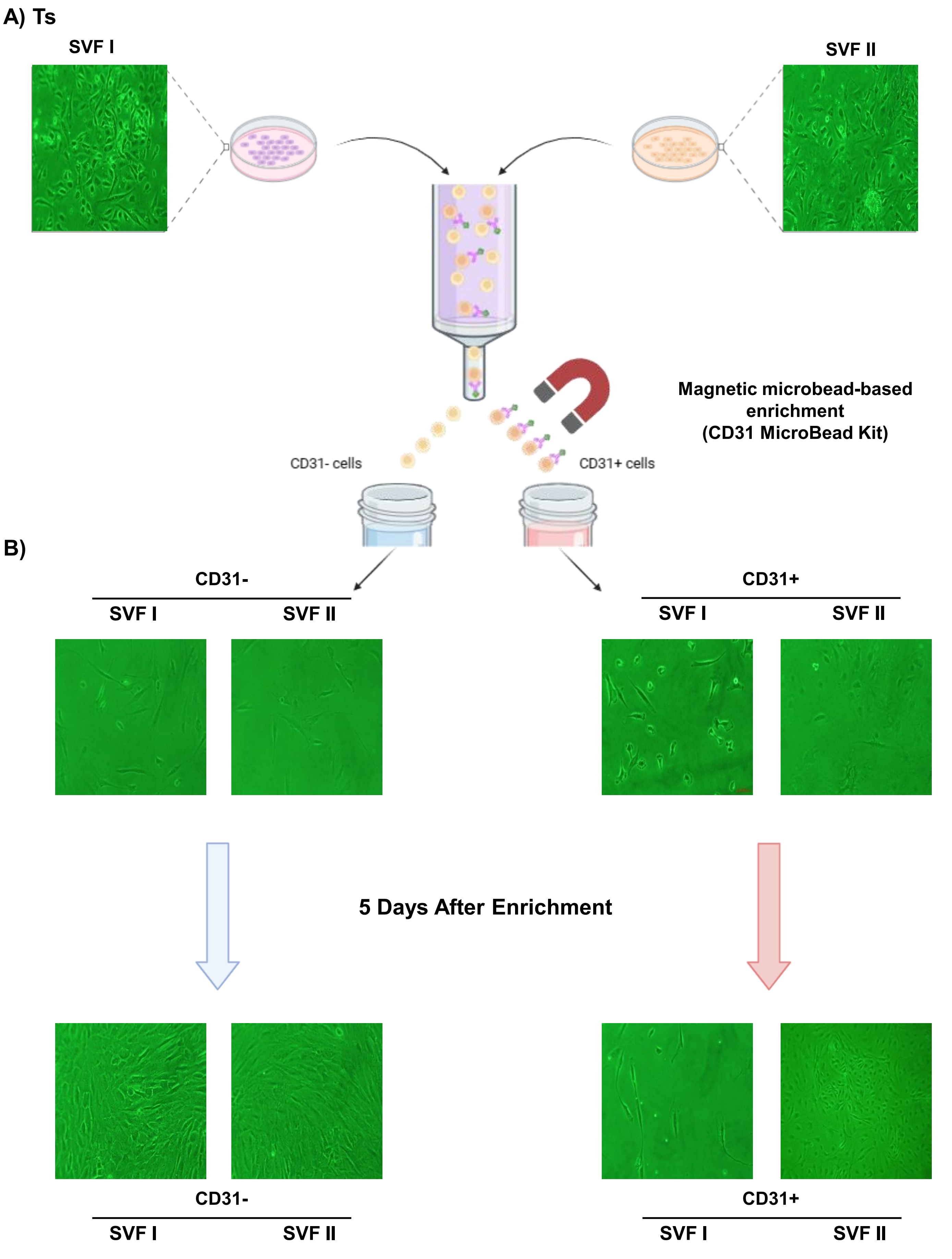

Supplementary Figure 2

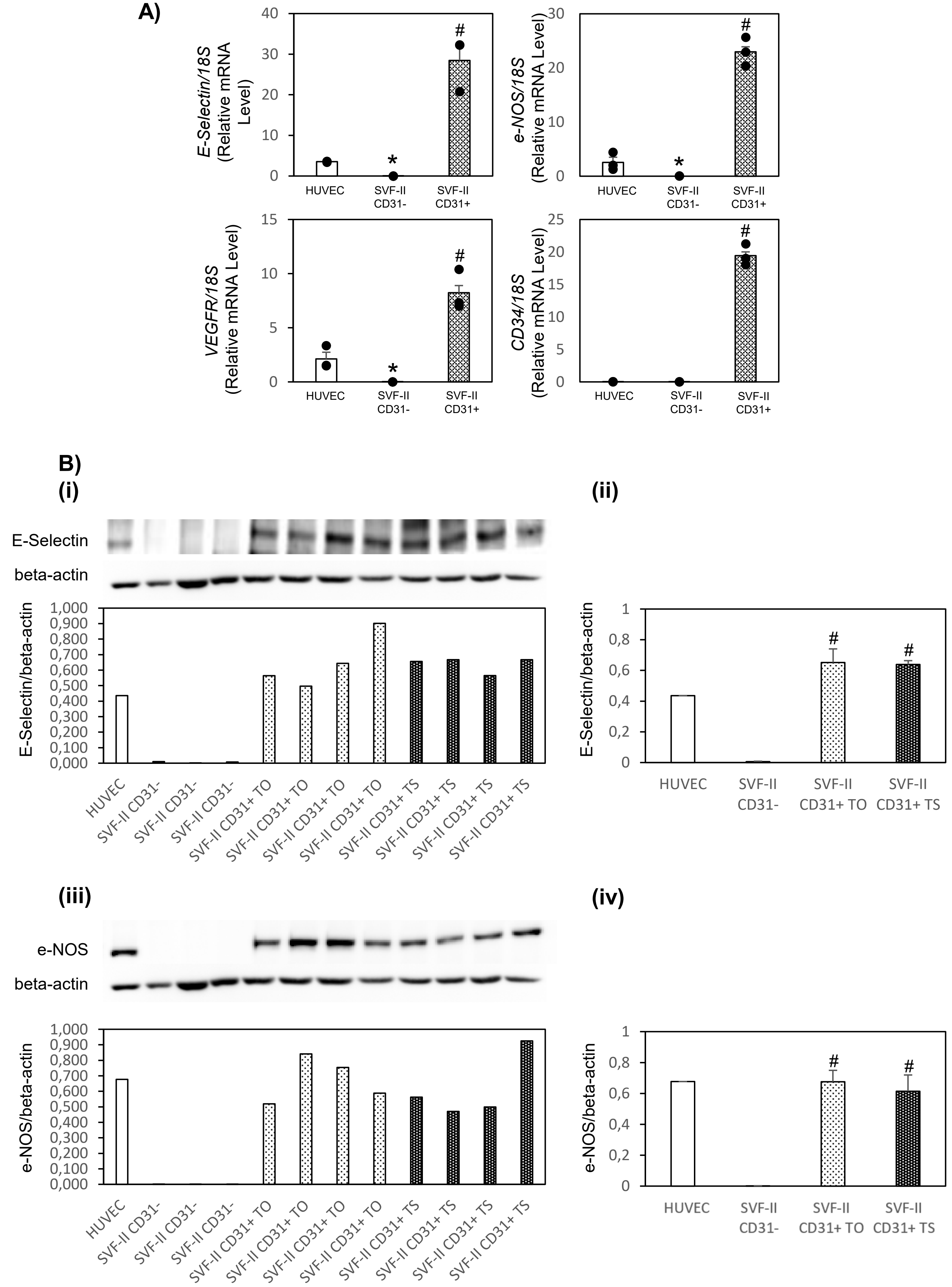

Supplementary Figure 3

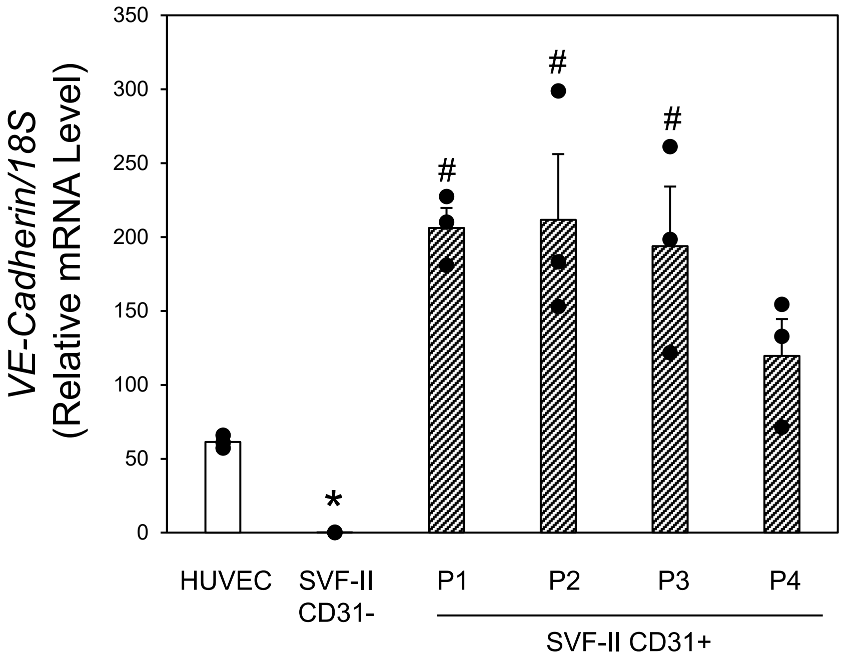

B)

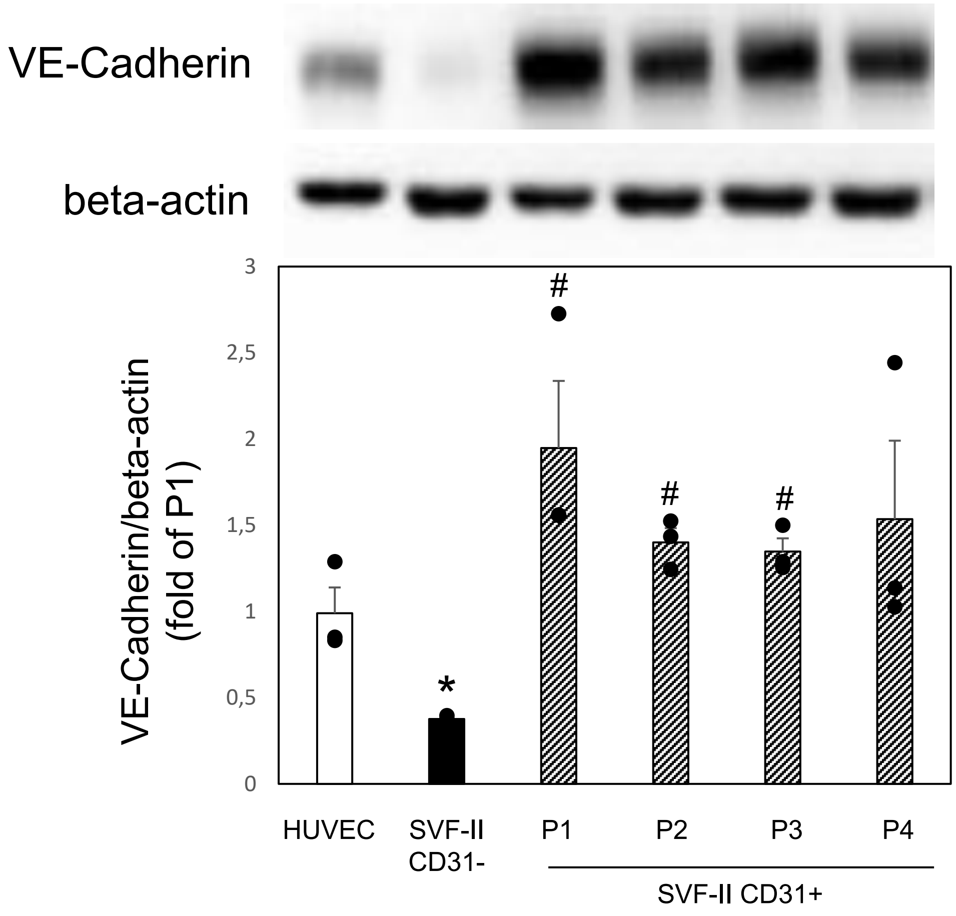

Supplementary Table 1

	**Obese (n = 20)**
**Sex (male/female)**	8/12
**Age (years)**	49.6 ± 9.7
**BMI (kg/m^2)**	44.7 ± 5.7
**Blood glucose (mg/dl)**	107.6 ± 16.0
**Fasting insulin (µUI/ml)**	33.7 ± 30.6
**HbA1c %**	5.6 ± 0.4
**HOMA-IR**	9.8 ± 10.2
**Total cholesterol (mg/dl)**	216.6 ± 38.6
**HDL cholesterol (mg/dl)**	49.2 ± 15.4
**Fasting triglycerides (mg/dl)**	182.5 ± 94.7
**Systolic pressure (mmHg)**	137.9 ± 17.7
**Diastolic pressure (mmHg)**	81.6 ± 7.5
**WBC (10^3 µl)**	10.1 ± 2.5

Supplementary Table 2

PRIMER	***SEQUENCE (5′-3′)***
E-Selectin For	GAAGGATGGACGCTCAATGG
E-Selectin Rev	TGGACTCAGTGGGAGCTTCAC
eNOS For	GGCCAACGCCGTGAAG
eNOS Rev	TCGGAGCCATACAGGATTGTC
VEGFR For	GTGACCAACATGGAGTCGTG
VEGFR Rev	CCAGAGATTCCATGCCACTT
CD34 For	CATCACAGAAACGACAGTCAA
CD34 Rev	ACTCCGCACAGCTGGAGG
VE-Cadherin For	CAGCCCAAAGTGTGTGAGAA
VE-Cadherin Rev	CGGTCAAACTGCCCATACTT
18S For	CGAACGTCTGCCCTATCAACTT
18S Rev	ACCCGTGGTCACCATGGTA

Supplementary Figure 1. **Magnetic microbead-based enrichment of CD31+ cells from SVF-I and SVF-II isolated from subcutaneous tissue. A)** SVF-I and SVF-II underwent magnetic microbead-based enrichment based on CD31, and both positive and negative fractions were observed by using light microscopy. **B)** CD31+ and CD31- cells from enrichment based on CD31 of SVF-I and SVF-II fractions were cultured, and after 5 days endothelial-like colonies with typical cobblestone morphologies were present only in the CD31+ cell cultures obtained from SVF-II; conversely, CD31+ cells were almost absent in SVF-I. CD31- cells from SVF-I and SVF-II exhibited a fibroblast-like morphology. Scale bar, 10 μm. SVF, stromal vascular fraction.

Supplementary Figure 2. Expression of specific endothelial cell markers in CD31+ and CD31- cells from SVF-II isolated from subcutaneous tissue. A) E-selectin, e-NOS, VEGFR, and CD34 mRNAs were analyzed in CD31+ and CD31- cells from SVF-II by quantitative reverse transcription PCR. All data are presented as mean ± standard error of the mean of three experiments, which were carried out using cells from different human donors. *, $p<0.05$ vs HUVECs; #, $p<0.05$ vs CD31- cells. HUVECs were used as positive control. 18S was used as loading control. **B)** E-selectin and e-NOS proteins were analyzed in CD31- cells from SVF-II and in CD31+ cells from SVF-II obtained from omental or subcutaneous tissue by immunoblotting. Figures B-i and B-iii show data obtained from different human donors; in figures B-ii and B-iv data are presented as mean ± standard error of the mean of experiments which were carried out using cells from different human donors. #, $p<0.05$ vs CD31- cells. HUVECs were used as positive control. Beta-actin was used as loading control. HUVECs, human umbilical vein endothelial cells; SVF, stromal vascular fraction.

Supplementary Figure 3. Evaluation of VE-Cadherin mRNA and protein expression in CD31+ cells from SVC-II cultured up to the fourth cell passage. VE-Cadherin, mRNA and protein expression, was evaluated in CD31+ cells from SVC-II cultured up to the fourth cell passage, by quantitative real-time PCR and immunoblotting, respectively. All data are presented as mean ± standard error of the mean of three experiments. *, $p<0.05$ vs HUVECs; #, $p<0.05$ vs CD31- cells. HUVECs were used as positive control. CD31- cells were used as negative control. HUVECs, human umbilical vein endothelial cells; SVC, stromal vascular cells.

Supplementary Table 1. Characteristics of the patients from whom the omental adipose tissue was obtained for endothelial cell isolation.

Supplementary Table 2. Primer sequences used for quantitative reverse transcription PCR.
